# Safety Assurance Factors for Electronic Health Record Resilience (SAFER): study protocol

**DOI:** 10.1186/1472-6947-13-46

**Published:** 2013-04-12

**Authors:** Hardeep Singh, Joan S Ash, Dean F Sittig

**Affiliations:** 1 Houston VA HSR&D Center of Excellence, the Michael E. DeBakey Veterans Affairs Medical Center and the Section of Health Services Research, Department of Medicine, Baylor College of Medicine, 2002 Holcombe Blvd, Houston, TX 77030, USA; 2 Department of Medical Informatics and Clinical Epidemiology, School of Medicine, Oregon Health & Science University, Portland, OR, USA; 3 University of Texas School of Biomedical Informatics and the UT-Memorial Hermann Center for Healthcare Quality & Safety, Houston, TX, USA

**Keywords:** Electronic health records, Health information technology, Patient safety, Risk assessment, Resilience

## Abstract

**Background:**

Implementation and use of electronic health records (EHRs) could lead to potential improvements in quality of care. However, the use of EHRs also introduces unique and often unexpected patient safety risks. Proactive assessment of risks and vulnerabilities can help address potential EHR-related safety hazards before harm occurs; however, current risk assessment methods are underdeveloped. The overall objective of this project is to develop and validate proactive assessment tools to ensure that EHR-enabled clinical work systems are safe and effective.

**Methods/Design:**

This work is conceptually grounded in an 8-dimension model of safe and effective health information technology use. Our first aim is to develop self-assessment guides that can be used by health care institutions to evaluate certain high-risk components of their EHR-enabled clinical work systems. We will solicit input from subject matter experts and relevant stakeholders to develop guides focused on 9 specific risk areas and will subsequently pilot test the guides with individuals representative of likely users. The second aim will be to examine the utility of the self-assessment guides by beta testing the guides at selected facilities and conducting on-site evaluations. Our multidisciplinary team will use a variety of methods to assess the content validity and perceived usefulness of the guides, including interviews, naturalistic observations, and document analysis. The anticipated output of this work will be a series of self-administered EHR safety assessment guides with clear, actionable, checklist-type items.

**Discussion:**

Proactive assessment of patient safety risks increases the resiliency of health care organizations to unanticipated hazards of EHR use. The resulting products and lessons learned from the development of the assessment guides are expected to be helpful to organizations that are beginning the EHR selection and implementation process as well as those that have already implemented EHRs. Findings from our project, currently underway, will inform future efforts to validate and implement tools that can be used by health care organizations to improve the safety of EHR-enabled clinical work systems.

## Background

Several countries have made recent multi-billion dollar investments in electronic health record (EHR) infrastructure to transform their health care delivery systems. However, implementation of EHR-related initiatives has encountered greater than expected challenges [1-4]. Although successful transformations have occurred in a few pioneering healthcare organizations across the globe, [5,6] the vast majority of organizations are still in the process of implementing their EHRs and modifying their work processes [7,8].

In some instances, reports warn of unintended consequences of health information technology (HIT) adoption, including new safety problems and reduced provider efficiency resulting from the implementation and use of EHRs [9-20]. EHR-related errors occur in a sociotechnical environment, described more fully below, which includes the hardware and software required to implement the heath IT, as well as the social environment in which it is implemented. EHR-related errors should be defined from the sociotechnical viewpoint of end users, rather than from the purely technical viewpoint of manufacturers, developers, vendors, and personnel responsible for implementation. In this context, EHR-related errors could occur anytime the EHR is unavailable for use, malfunctions, is used incorrectly, or when EHR components interact incorrectly, resulting in data being lost or incorrectly entered, displayed, or transmitted [20]. Examples could include technology errors, such as lack of transmission of test results due to software configuration problems, or technology use problems such as juxtaposition errors due to clicking the wrong item in a drop-down menu when ordering a medication. EHR-related errors are complex, and the roots of these errors are often multifaceted. Risks for EHR-related errors and breakdowns may be related to features of the technology itself, user behaviors, and organizational influences on how the EHR is routinely used, maintained, and monitored.

To respond to the safety challenges described herein, the Office of the National Coordinator for Health Information Technology (ONC) sponsored an Institute of Medicine report, *Health IT and Patient Safety: Building Safer Systems for Better Care*[21]. In addition, ONC leveraged another ongoing project on “Anticipating the Unintended Consequences of Health IT” and requested development of health IT patient safety self-assessment guides to address these safety concerns.

To ensure that EHRs fulfill their promise of making healthcare safer and enhancing health care quality, hospitals and other clinical entities require proactive monitoring strategies to detect new, unexpected EHR-related errors. This would enable them to transform into safe and effective “EHR-enabled clinical work systems” by building resilience into their systems and processes. Resilience is the “degree to which a system continuously prevents, detects, mitigates or ameliorates hazards or incidents so that an organization can bounce back to its original ability to provide care” [22]. Through proactive, systematic assessment of risks and vulnerabilities, health care organizations can address potential EHR-related safety hazards before harmful incidents occur. The overall objective of this project is to develop and validate proactive self-assessment tools to ensure that EHR-enabled clinical work systems are safe and effective.

## Methods/Design

The project will be conducted in two main phases. The first phase develops self-assessment guides that can be used by clinicians and health care organizations to evaluate certain high-risk components of their EHR-enabled clinical work systems. The second phase will examine the usefulness of the self-assessment guides by beta testing the guides at selected sites with EHR-enabled systems and by conducting on-site evaluations.

**Figure 1:**
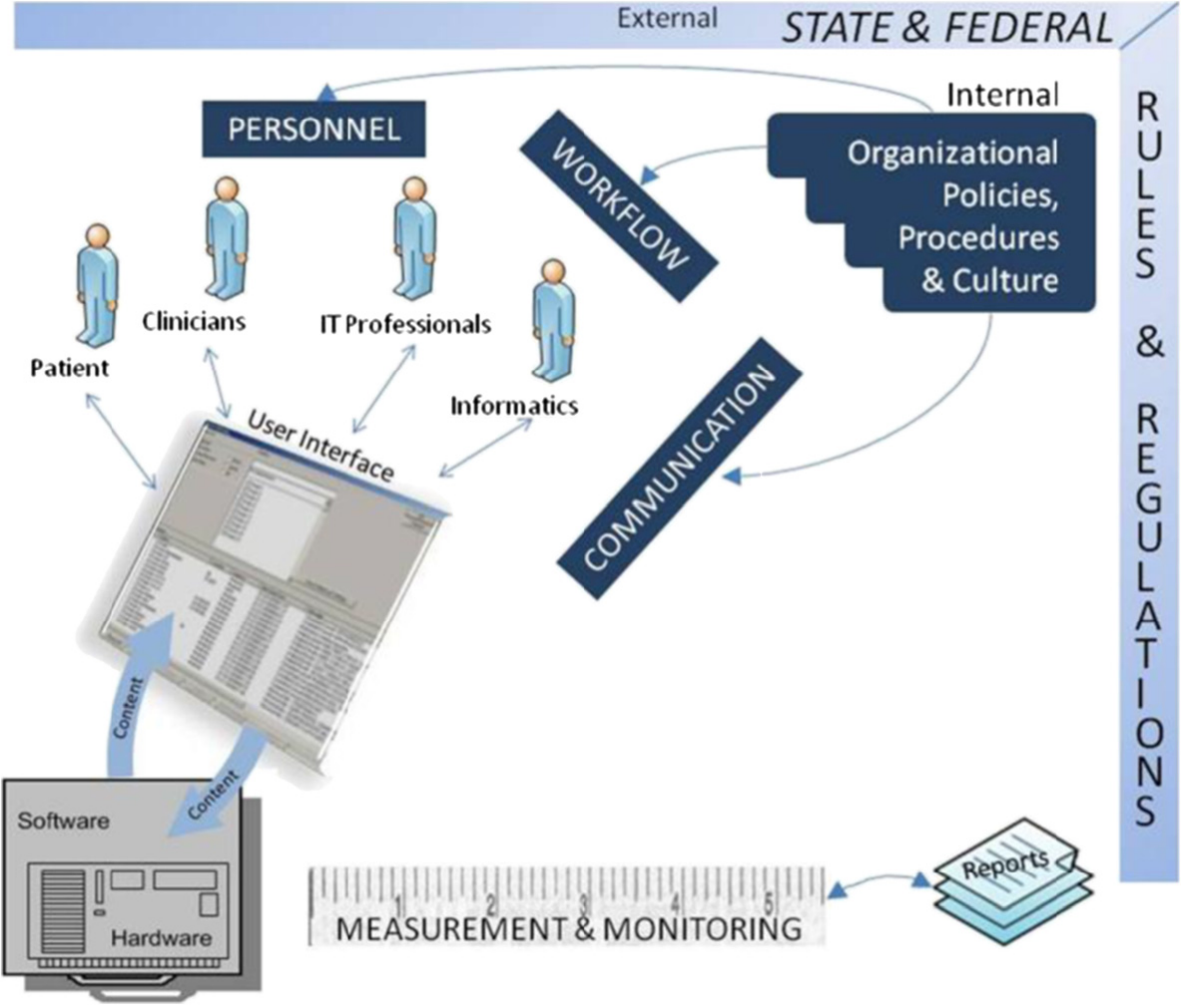
**Sociotechnical model for safe and effective health IT use (reproduced with permission from BMJ Quality and Safety)**[23]**.**

This work will be guided by an 8-dimension, sociotechnical model of safe and effective health IT use [23] that we developed in response to difficulties clinicians and organizations have encountered with EHR implementation. This model (see Figure 1 and Table 1) provides a comprehensive framework for studying all aspects of health IT design, development, implementation, use, and evaluation within complex, adaptive health care systems.

**Table 1:** **Dimensions of the sociotechnical model**[23]


*Hardware and software*	The computing infrastructure used to power, support, and operate clinical applications and devices.
*Clinical content*	The text, numeric data, and images that constitute the “language” of clinical applications.
*Human-computer interface*	All aspects of technology that users can see, touch, or hear as they interact with it.
*People*	Everyone who interacts in some way with technology, including developers, users, IT personnel, and informaticians.
*Workflow and communication*	Processes to ensure that patient care is carried out effectively.
*Internal organizational features*	Policies, procedures, work-environment and culture.
*External rules and regulations*	Federal or state rules that facilitate or constrain preceding dimensions
*Measurement and monitoring*	Processes to evaluate both intended and unintended consequences of health IT implementation and use.

We will use the eight dimensions of this sociotechnical model (Figure 1) to develop self-assessment guides to address certain high-risk aspects of the EHR-enabled clinical work system. These dimensions include the *hardware and software* that “run” the EHR; the *clinical content* that is used to configure the various EHR modules; the *user interface* that allows clinicians to interact with the EHR application; the *people* who are required to configure the system, train the users, and use the system; the *clinical workflow and communication processes* that enable clinicians to provide patient care; the *internal organizational policies, procedures, and culture* that “govern” all the activities associated with using the EHR; the *external rules and regulations* that affect the healthcare delivery system; and, finally, the *measurement and monitoring* that is required to determine what is working and what is not. Within each of these 8 dimensions we will identify multiple themes that affect safety and effectiveness of EHR-enabled work systems in order to provide guidance about what an organization could do to address potential problem areas.

Based upon previous work, current literature, and expert opinion, [1,3,14,15,24-29] we have identified 9 high-risk areas around which the content of the self-assessment guides will be organized. These areas include features, functions, or applications of the EHR itself and/or relate to patient safety issues that can arise from the use of the EHR. The 9 high-risk areas are:

•Computerized provider order entry (CPOE) and e-prescribing

•Clinical decision support

•Test result reporting

•Communication between providers

•Patient identification

•EHR downtime events

•EHR customization and configuration

•System-system interface data transfer

•Health IT safety-related human skills

### Procedures for developing the self-assessment guides

We will develop guides in each of the 9 high-risk areas identified above through a stepwise process of consultation and data gathering with subject matter experts as well as stakeholders with specific interests in quality improvement and patient safety. These steps are listed below.

Step 1: Convening an expert panel and workgroup

We will identify and recruit an expert panel and a technical workgroup, each consisting of approximately 4–6 experts, to provide guidance and advice on several aspects of the project including design of the self-assessment guides, facilitating access to key stakeholders and organizations that might ultimately use these guides, and facilitating access to institutions that would be willing to serve as test sites. The areas of expertise to be represented on the expert panel will include EHRs, patient safety, quality improvement, risk management, human factors engineering and usability, knowledge and experience with small physician office practices, and accreditation and certification practices for patient safety. The technical workgroup will include clinical and technical experts who can help develop and prioritize specific components of each of the guides to be developed.

The expert panel will meet in person once in the early phase of the project. The goal of this meeting will be to inform members about this project, help select study sites, identify which individuals will need to be interviewed during site visits, and identify the best strategies to develop the guides and implement them in the field. Over the course of the project, the expert panel will convene twice more through two teleconferences, which will be used to review and refine the tools and to work further on strategies to get the guides into the field.

The technical workgroup will hold one teleconference and will be available to review the guides as needed. The goal of the teleconference will be to discuss and prioritize initial lists of items in the guides, and the reviews will be focused on recommendations for modifying the items after site visits. For expert panel and technical workgroup meetings, both modified Delphi and normative group techniques will be used for assessing content validity for the guides. Between meetings we expect to obtain additional feedback on guide items via e-mail. The following is a preliminary list of questions that would be asked:

Question 1-Content: Do you consider the items of high enough importance to be included in this guide, and are there themes or items that we have not considered which must be included?

Question 2-Wording/comprehension: Are the items appropriately worded so that a multidisciplinary team assembled by the organization conducting this as a "self-assessment" (including representatives from groups such as Chief Medical Informatics Officers (CMIOs), Chief Information Officers (CIOs), Chief Nursing Informatics Officers, risk management/quality and safety personnel, ambulatory clinic practice managers, and specialized personnel in pharmacy/lab etc.) is able to answer them and find them useful for improvement?

Question 3-Prioritization: Are there some items on the checklist that you would consider “essential” for all organizations regardless of size, EHR vendor, type of organization, etc.?

Step 2: Literature search

We will conduct an updated literature search to inform the development of the self-assessment guides. Because this field is fairly nascent, sources from the web and press reports on EHR safety events will be considered in addition to the peer-reviewed scientific literature.

Step 3: Stakeholder engagement for development and future dissemination

It will be important to engage with, educate, and solicit feedback from key stakeholders other than the expert panel and technical workgroup members to ensure that the final guides are supported and their use encouraged for the target audiences many of these stakeholders represent. Given the small membership of the expert panel and technical workgroup, stakeholder engagement will also serve to broaden the awareness level of many more influential individuals and organizations about issues related to HIT safety. The key stakeholders invited to participate will vary depending on the topic area for each guide. We will involve some in developing the guides, some in gaining their endorsement of the guides, and others in actually using the guides. We will not reimburse stakeholders for participation in these activities, except for individuals who represent stakeholder groups who might also serve on expert panels.

We will make initial contacts by e-mail and phone. Some discussions will require conference calls. We will prepare explanatory materials for written and oral presentation that include a description of the problem we are trying to address and how this project will address it. Members of our team will also attend conferences where we can give presentations about the objectives of the project and, later, to describe results of our work and solicit suggestions.

Step 4: Development of the self-assessment guides

We will develop the guides as self-contained chapters or modules, each devoted to one of the 9 different high-risk areas. The guides themselves will be created in the form of question lists with accompanying instructions for completion and an introduction explaining the methodology for developing each guide.

Our multi-disciplinary team of clinicians, health services researchers, human factors engineers, computer scientists, and informaticians will develop and continuously refine on an iterative basis, an itemized list of characteristics of reliability of safety and effectiveness within each of the 8 dimensions. For example, within the hardware/software dimension, we have identified the need for redundant hardware for key system components (e.g., database servers, and distributed clinical workstations) as an important safety feature. Likewise, within the workflow and communication dimension, we have identified methods of promptly notifying clinicians that new, abnormal test results are available as an important characteristic. We expect to initially identify about 7–10 items within each of the 8-dimensions of our sociotechnical model for each of the guides. The initial list will be narrowed down using the steps above.

This development process will incorporate the input of key stakeholders, our expert panel, and the technical workgroup as described above. Lists of items for portions of each tool will be reviewed and prioritized in the most appropriate manner for that module. Once the guides are drafted, we will pilot test them using interviews with intended end users at local facilities.

Step 5: Face validation and refinement of the guides

This step will involve 5 site visits to healthcare organizations for two purposes. First, we will establish the content validity of the checklists with a group of likely users. For example, we will ask individuals to answer the questions posed by the guides and solicit feedback about the questions themselves and the perceived usefulness of the guides. The second purpose of these visits will be to assess the context within which the tools could be used. Data collection will include semi-structured and informal interviews, naturalistic observation of various processes and people, and document analysis. Interview subjects will be selected for their expertise and roles in HIT safety. Observation subjects will represent a cross-section of system users including both “EHR skeptics” and champions. Our team has previously used these methods to understand operations of EHR work systems [30-32]. Our team members from multiple disciplines will be present at the site visits.

The site visits will be essential to understanding the local contexts and constraints in which EHRs are being implemented, as these are expected to be highly variable even within the same organization. The data we obtain will shed light on who would most likely use the guides and how. We will also use the site visits to identify high-risk areas that site leaders believe to be associated with safety and effectiveness problems. Site selection for the 5 sites will be based on geography, size, and type (e.g., academic medical center, privately-owned out-patient clinics, EHR service providers) of organization. We will visit sites with variable types of EHR-related characteristics such as 1) experience with using clinical information systems, 2) comprehensiveness of approach and/or resources to address EHR safety, and 3) size and type of institution (ambulatory vs. inpatient). The tools will be modified based on the information gathered, and later they will be beta tested at other sites to establish the integrity of our findings.

### Procedures for beta testing the self-assessment guides

Each of the 9 guides will be beta tested at five additional sites. Two site visitors will interview key informants at each site for feedback and recommendations about further improvement. On-site evaluation will also help us understand how local site assessors will interpret the items and determine what is considered feasible and useful for the organizations to accomplish. We will work with IT professionals, clinicians, and other personnel to compare each item within our self-assessment guides to acknowledged industry-standard best practices. For instance, items that are not found to be easily operationalized in terms of observations and/or measurements will be edited or removed, and new items will be added as needed. One representative from each site will assist our team and help us compare the organization’s responses to the items with our own assessments. This process will help ensure that the assessment guides are valid and that multiple respondents interpret items in the same way.

Although we anticipate that our initial development process will result in preliminary guides that have strong content and content validity, we will leave open the potential for substantial further refinement during the beta testing period. We will approach the refinement of the guides iteratively such that the guides will be modified after each visit and new versions tested at the next site.

The anticipated output of this work will be a set of highly generalizable self-administered EHR assessment guides that transform the final set of items into clear checklist-type items with a set of discrete responses. Each item will include an explanation of its relevance and clear descriptions of response choices. Our future goal is for these guides to automatically generate a narrative report on findings and conclusions and, ultimately, provide a comparison to benchmark results. At the end of the beta testing and refinement phase, the guides will be ready for field testing at additional sites. However, true validation testing is outside the scope of this project.

## Discussion

We propose to develop self-assessment guides that health care organizations can use to help prevent, detect, mitigate, and ameliorate hazards associated with the use of EHRs. Our project is grounded conceptually in a multifaceted sociotechnical model of safe and effective use of health IT. In building upon the work proposed here, future initiatives can help create best practices that can be used by key stakeholders to oversee the successful transformation of their health care system into a highly reliable EHR-enabled clinical work system [29].

**Figure 2:**
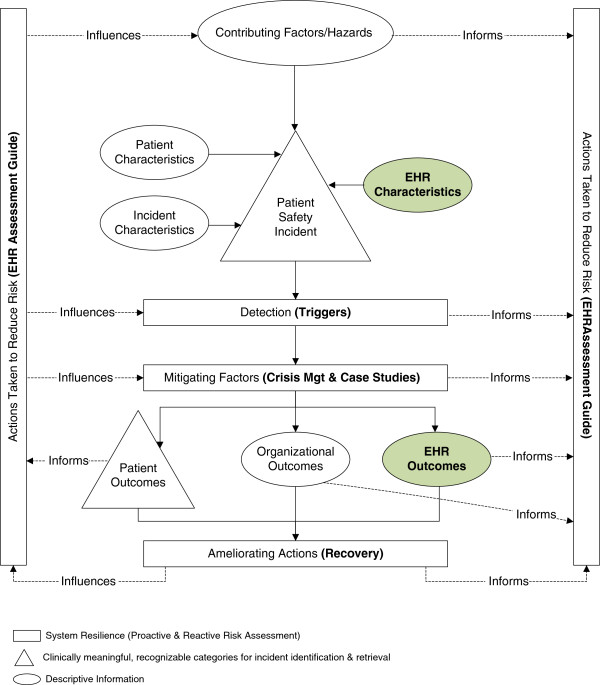
Modified conceptual model showing relationships between patient safety and resilience within an EHR-enabled work system.

The development of proactive risk assessment guides is consistent with the WHO conceptual model for patient safety (see Figure 2). The WHO model focuses on several aspects of system resilience. For example, we will aim to develop guides that help users not only detect risks but also develop “actions or [create] circumstances which prevent or moderate the progression of an incident toward harming the patient” [33]. Users can in turn learn from the ameliorating actions for the event, i.e., identify the changes that were made, and assess the outcomes associated with the patient, organization, and the EHR. Based on this knowledge, organizations can identify and further develop specific actions that will reduce the risk that the error will occur in the future.

Any broad strategy to address patient safety activities within EHR-enabled health care systems must account for variation in stages of EHR implementation and levels of complexity across clinical practice settings. For instance, organizations that have recently deployed or are still planning to implement EHRs may face different challenges to ensuring patient safety (e.g., intraoperability between the EHR and other systems) than their counterparts with established EHRs (e.g., making full use of the EHR to monitor safety events). Thus, implementing proactive assessment of EHR risks could be conceptualized in three phases. The first phase would address safety concerns unique to technology (i.e., ensuring that basic EHR functions are safe and reliable). The second phase would address mitigation of safety concerns from failure to use technology appropriately (i.e. safer application and use of EHRs). In the third phase, organizations will leverage EHR capabilities to monitor and improve patient safety [29]. The assessment is expected to provide input to help organizations or practices to identify and prioritize patient safety issues related to most aspects of EHR-enabled health care delivery. On the basis of this information, they could pursue additional strategies and risk management tools to address the EHR-related patient safety risks identified and engage their leadership in this process.

Our assessment strategies also pose some limitations and challenges. This assessment will require input and time from a number of different individuals, which could include IT managers (e.g., CIO or CMIO), risk managers, practice managers, patient safety and quality personnel, as well as other key stakeholders that are involved in ensuring the safety of an EHR-enabled health care system (nursing, pharmacy, laboratory personnel, and others). Although we will recommend a multidisciplinary team to work together to complete this assessment, this might not always be achievable with internal personnel in every setting. For instance, in small organizations, outside IT expertise will often be required. In some instances, the involvement of the IT vendor/developer may be required. Furthermore, when evaluating specific safety issues that may involve several interacting factors within the sociotechnical model, even large organizations may choose to involve outside organizations or personnel with specific expertise in understanding and addressing health-IT related concerns.

Risk assessment based upon these guides will also be highly context dependent. Thus, the risks associated with not fully implementing practices identified in the health IT self-assessment guides will vary and should be considered within the context of each individual setting. For example, an EHR downtime poses different risks for a small ambulatory practice than it does for a 500-bed level one trauma hospital with an active emergency department and several intensive care units. Settings will need to consider both the *severity* and the *probability* (i.e., in terms of frequency) of a safety event that might result from not implementing practices identified in these guides.

EHRs are changing the way we deliver health care. Taken together, error detection, mitigation, and amelioration are the three most important concepts in building system resiliency to reduce the risk of future safety events within the EHR-enabled work system. Lessons learned from the development of the assessment guides will be helpful for both organizations that are beginning the EHR selection and implementation process as well as those that have already implemented systems. The health IT patient safety assessment guides might lead institutions to better leverage the benefits of EHRs. Using a multi-faceted, cross-disciplinary approach to develop evidence-based guidance for the EHR-enabled work system might be a useful step in improving patient safety in technology-enabled health care.

## Competing interests

HS, JSA, and DFS have no competing interests to declare.

## Authors’ contributions

HS, JSA, and DFS drafted the protocol. All authors made substantial contributions to the design of the study and contributed feedback on the protocol. HS drafted the manuscript, to which all authors provided feedback and final approval.

## Pre-publication history

The pre-publication history for this paper can be accessed here:

http://www.biomedcentral.com/1472-6947/13/46/prepub
